# Translational Bioinformatics for Diagnostic and Prognostic Prediction of Prostate Cancer in the Next-Generation Sequencing Era

**DOI:** 10.1155/2013/901578

**Published:** 2013-07-15

**Authors:** Jiajia Chen, Daqing Zhang, Wenying Yan, Dongrong Yang, Bairong Shen

**Affiliations:** ^1^Center for Systems Biology, Soochow University, Suzhou 215006, China; ^2^School of Chemistry, Biology and Material Engineering, Suzhou University of Science and Technology, Suzhou 215011, China; ^3^Department of Urology, The Second Affiliated Hospital of Soochow University, Suzhou 215004, China

## Abstract

The discovery of prostate cancer biomarkers has been boosted by the advent of next-generation sequencing (NGS) technologies. Nevertheless, many challenges still exist in exploiting the flood of sequence data and translating them into routine diagnostics and prognosis of prostate cancer. Here we review the recent developments in prostate cancer biomarkers by high throughput sequencing technologies. We highlight some fundamental issues of translational bioinformatics and the potential use of cloud computing in NGS data processing for the improvement of prostate cancer treatment.

## 1. Introduction

Prostate cancer (PCa) is the most common cancer and the second leading cause of cancer deaths among males in western societies [1]. It is estimated that 241740 new PCa cases were diagnosed and that 28170 men died from it in the United States in 2012. Since its discovery over 20 years ago, Prostate Specific Antigen (PSA) has been the mainstay for diagnosis and prognosis of prostate cancer. However, the routine use of PSA screening remains controversial, owing to its limited specificity. PSA fails to differentiate PCa from common prostate disorders; moreover, it cannot discriminate between aggressive tumors and low-risk ones that may otherwise never have been diagnosed without screening [2]. As such, overdetection and overtreatment represent critical consequences of PSA-based screening [3]. The ongoing debate highlights the need for more sensitive and specific tools to enable more accurate diagnosis and prognosis. 

During the last decade, the ability to interrogate prostate cancer genomes has rapidly advanced. The resolution for genomic mutation discovery was improved first with array-based methods and now with next-generation sequencing (NGS) technologies. These high throughput technologies open up the possibility to individualize the diagnosis and treatment of cancer. However significant challenges, particularly with respect to integration, storage, and computation of large-scale sequencing data, will have to be overcome to translate NGS achievements into the bedside of the cancer patient. Translational informatics evolves as a promising methodology that can provide a foundation for crossing such “translational barriers” [4, 5].

 Here we overview the NGS-based strategies in prostate cancer research, with focus on upcoming biomarker candidates that show promise for the diagnosis and prognosis of prostate cancer. We also outline future perspectives for translational informatics and cloud computation to improve prostate cancer management.

## 2. Microarray Based Diagnosis and Prognosis of PCa

In the past two decades, high-throughput microarray profiling has been utilized to track complex molecular aberrations during PCa carcinogenesis. We performed a comprehensive search in the Gene Expression Omnibus (GEO) for the array-based profiles in human PCa. The retrieved GEO series generally fall into 5 categories: gene expression profiling, noncoding RNA profiling, genome binding/occupancy profiling, genome methylation profiling, and genome variation profiling. The number of GEO series for each category is summarized in Table 1. 

Together these array-based technologies have shed light on the genetic alterations in the PCa genome. Among the abnormalities affecting prostate tumors, the copy number alteration is the most common one [6].

Numerous early studies have used comparative genome hybridization (CGH) or single nucleotide polymorphism (SNP) arrays to assess copy number changes in tumor DNA. As a result, multiple genomic regions that displayed frequent gain or loss in the PCa genome [6–11] have been revealed. Chromosome 3p14, 8p22, 10q23, 13q13, and 13q14 are found to display broad copy number deletion. Key genes mapping within these deleted regions include NKX3.1, PTEN [12], BRCA2, C13ORF15, SIAH3 [11], RB1, HSD17B2 [9], FOXP1, RYBP, and SHQ1 [6]. High-level copy number gains are detected at 5p13, 14q21, 7q22, Xq12, and 8q13 [9]. Key amplified genes mapping within these regions include SKP2, FOXA1, AR [11], and HSD17B3 [9].

Using microarray, substantial efforts have also been made to characterize prostate cancer gene expression profiles. Differentially expressed genes identified in these studies point to a plethora of candidate biomarkers with diagnostic or prognostic value. 

A diagnostic marker is able to differentiate prostate cancer with other prostatic abnormalities. There are many emerging markers that show promise for PCa diagnosis, such as alpha-methylacyl-CoA racemase (AMACR) [13], prostate cancer gene 3 (PCA3) [14], early prostate cancer antigen (EPCA)-2 [15], Hepsin [16], kallikrein-related peptidase 2 (KLK2) [17], and polycomb group protein enhancer of zeste homolog 2 (EZH2) [18]. The most prominent of these is PCA3, which was found to exhibit higher sensitivity and specificity for PCa detection than PSA. PCA3 thus provides a potential complement to PSA for the early diagnosis of PCa.

Prognostic biomarkers for prediction of prostate cancer patient outcome have also been identified. Increased serum levels of IL-6 and its receptor (IL-6R) are associated with metastatic and hormone refractory disease [19]. Elevated serum chromogranin A levels are indicative of poor prognosis and decreased survival [20]. Other differentially expressed molecules with prognostic potential include the urokinase plasminogen activation (uPA) [21], TGF-*β*1 [22, 23], MUC1 [24], CD24 [25], hCAP-D3 [26], vesicular monoamine transporter 2 (SLC18A2) [27], TEA domain family member 1 (TEAD1), c-Cbl [28], SOX7 and SOX9 [29], nuclear receptor binding protein 1 (NRBP1) [30], CD147 [31], and Wnt5a [32]. Each of these markers will require proper validation to ensure their clinical utility.

While microarray technology represents a wonderful opportunity for the detection of genomic alterations, there are significant issues that must be considered. For example, array-based methods are impossible to detect variations at a low frequency (many well below 1%) in the samples. In addition, microarrays can only provide information about the genes that are already included on the array. The emerging next-generation sequencing technology, also called massively parallel sequencing, however, helps to overcome the challenges by generating actual sequence reads [33].

## 3. NGS Based Diagnosis and Prognosis of PCa

Key feature of the next-generation sequencing technology is the massive parallelization of the sequencing process. By virtue of the massively parallel process, NGS generates hundreds of millions of short DNA reads (100–250 nucleotides) simultaneously, which are then assembled and aligned to reference genomes.

A number of NGS systems are available commercially, including Genome Analyzer/HiSeq 2000/MiSeq from Illumina, SOLiD/PGM/Proton from Life Sciences, GS-FLX (454)/GS Junior from Roche, as well as novel single molecule sequencers, for example, Heliscope from Helicos Biosciences and SMRT offered by Pacific Biosciences. All these technologies provide digital information on DNA sequences and make it feasible to discover genetic mutations at unprecedented resolution and lower cost. 

**Figure 1:**
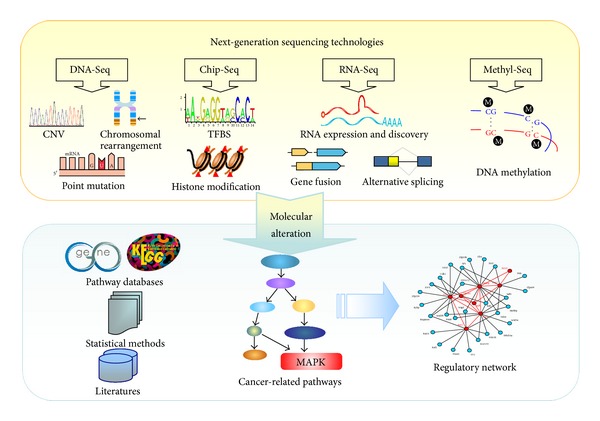
NGS-based pipeline for cancer marker discovery.

It is generally accepted that cancers are caused by the accumulation of genomic alterations. NGS methods can well squeeze all the alteration information that remains hidden within the genome, including point mutations, small insertions and deletions (InDels), copy number alterations (CNV), chromosomal rearrangements, and epigenetic alterations. For these reasons NGS has become the method of choice for large-scale detection of somatic cancer genome alterations and is changing the way how cancer genome is analyzed. An NGS-based research pipeline for PCa biomarkers is given in Figure 1. 

Currently, next-generation sequencing is being applied to cancer genome study in various ways:

1. genome-based sequencing (DNA-Seq), yielding information on sequence variation, InDels, chromosomal rearrangements, and copy number variations,
2. transcriptome-based sequencing (RNA-Seq), yielding quantitative information on transcribed regions (total RNA, mRNA, or noncoding RNAs),
3. interactome-based sequencing (ChIP-Seq), yielding information on protein binding sequences and histone modification,
4. methylome-based sequencing (Methl-seq), yielding quantitative information on DNA methylation and chromatin conformation.

In the following part, we will introduce the main applications to next-generation sequencing of prostate cancer, using examples from the recent scientific literature (summarized in Table 2).

### 3.1. DNA-Seq

According to the proportion of the genome targeted, DNA-Seq is categorized into whole-genome sequencing and exome sequencing. The goal of whole-genome sequencing is to sequence the entire genome, not just coding genes, at a single-base resolution. By whole-genome sequencing, recent studies have provided detailed landscape of genomic alterations in localized prostate cancers [6, 11, 46, 68, 69]. The full range of genomic alterations that drive prostate cancer development and progression, including copy number gains and losses, single nucleotide substitutions, and chromosomal rearrangements, are readily identified.

#### 3.1.1. Copy Number Alteration

Most prostate cancers exhibit somatic copy number alterations, with genomic deletions outnumbering amplifications [6]. Early methods for copy number analysis involve fluorescence in situ hybridizations and array-based methods (CGH arrays and SNP arrays). More recently, NGS technologies have been utilized and offer substantial benefits for copy number analysis.

NGS used changes in sequencing depth (relative to a normal control) to identify copy number changes. The digital nature of NGS therefore allows accurate estimation of copy number levels at higher resolution. In addition, NGS can provide novel gene copy information such as homozygous and heterozygous deletions and gene amplifications, whereas traditional sequencing approaches cannot. For example, by next-generation sequencing of castrate-resistant prostate cancer (CRPC), Collins et al. [34] identified a homozygous 9p21 deletion spanning the MTAP, CDKN2, and ARF genes and deficiency of MTAP was suggested as an exploitable tumor target.

#### 3.1.2. Somatic Nucleotide Substitutions

While whole-genome sequencing provides the most comprehensive characterization of the cancer genome, it is the most costly. Alternatively, targeted sequencing approaches, such as exome sequencing, assemble multiple cancer genomes for analysis in a cost-effective manner. Whole-exome sequencing captures the coding exons of genes that contain the vast majority of disease causing mutations. Relative to structural alterations, point mutations are less common in prostate cancer [6, 70] and the average mutation rate was estimated at 1.4 Mb^−1^ in localized PCa [35] and 2.0 Mb^−1^ in CRPC [36].

Capillary-based exome sequencing has extensively been performed in localized PCa and CRPC, and a handful of oncogenic point mutations have been defined. Remarkably, Taylor et al. [6] performed focused exon resequencing in 218 prostate cancer tumors and identified multiple somatic alterations in the androgen receptor (AR) gene as well as its upstream regulators and downstream targets. For example, the AR coactivator NCOA2 and p300, the AR corepressor NRIP1/RIP140 and NCOR2/SMRT were found to harbor somatic mutations. Other genes including KLF6, TP53, AR, EPHB2, CHEK2, and ATBF1 [6, 71–74] have also been reported to harbor somatic mutations in localized prostate cancer.

Recently NGS is becoming increasingly routine for exome sequencing analysis. Whole-exome sequencing using next-generation sequencing (NGS) technologies compares all exon sequences between tumors and matched normal samples. Multiple reads that show nonreference sequence are detected as point mutations. In this way a number of driver mutations in prostate cancer have been uncovered. Robbins et al. [11] used NGS-based exome sequencing in 8 metastatic prostate tumors and revealed novel somatic point mutations in genes including MTOR, BRCA2, ARHGEF12, and CHD5. Kumar et al. [37] performed whole-exome sequencing of lethal metastatic tumors and high-grade primary carcinomas. They also observed somatic mutations in TP53, DLK2, GPC6, and SDF4. More recently Barbieri et al. [35] and Grasso et al. [36] systematically analyzed somatic mutations in large cohorts of prostate tumors. Barbieri et al. [35] investigated 112 primary tumor-normal pairs and revealed novel recurrent mutations in SPOP, FOXA1, and MED12. Grasso et al. [36] sequenced the exomes of 11 treatment-naive and 50 lethal CRPC and identified recurrent mutations in multiple chromatin- and histone-modifying genes, including MLL2 and FOXA1. These two studies also reported mutated genes (SPOP [35] and CHD1 [36]) that may define prostate cancer subtypes which are ETS gene family fusion negative.

Together these findings present a comprehensive list of specific genes that might be involved in prostate cancer and prioritize candidates for future study.

### 3.2. RNA-Seq

In addition to genome applications, NGS will also dramatically enhance our ability to analyze transcriptomes. Before NGS, microarrays have been the dominant technology for transcriptome analysis. Microarray technologies rely on sequence-specific probe hybridization, and fluorescence detection to measure gene expression levels. It is subject to high noise levels, cross-hybridization and limited dynamic range. Compared to microarrays, the emerging RNA-Seq provides digital gene expression measurements that offer significant advantages in resolution, dynamic range, and reproducibility. The goal of transcriptome sequencing is to sequence all transcribed genes, including both coding and noncoding RNAs. It is independent of prior knowledge and offers capacity to identify novel transcripts and mutations that microarrays could not achieve, such as fusion genes, noncoding RNAs, and splice variants.

#### 3.2.1. Gene Fusions

Recurrent gene fusion is a prevalent type of mutation resulting from the chromosomal rearrangements, which can generate novel functional transcripts that serve as therapeutic targets. Early studies relied on cytogenetic methods to detect chromosomal rearrangements. However this method is only applicable in cases of simple genomes and is vulnerable in complex genomes of epithelial cancers such as PCa.

Complete sequencing of prostate cancer genomes has provided further insight into chromosomal rearrangements in prostate cancer. NGS technologies, for example, paired-end sequencing approaches are sufficiently sensitive to detect break point crossing reads and are extremely powerful for the discovery of fusion transcripts and potential break points. 

The first major recurrent fusion to be identified in prostate cancer was discovered by Tomlins et al. using Cancer Outlier Profile Analysis (COPA) algorithm [38]. The fusion discovered places two oncogenic transcription factors from the ETS family (ETV1 and ERG) under control of the prostate-specific gene TMPRSS2.

While the TMPRSS2:ETV1 fusion is rare and occurs in 1–10% of prostate cancers [75], the TMPRSS2:ERG fusion is present in roughly half of prostate cancers and is the most common genetic aberration so far described in prostate cancer. Furthermore TMPRSS2:ERG is unique only to prostatic nonbenign cancers [76]. Given this high specificity, its clinical application as ancillary diagnostic test or prognostic biomarker is promising. The expression of TMPRSS2:ERG fusion gene has been proposed as a diagnostic tool, alone or in combination with PCA3 [77]. In addition, many studies have suggested that TMRSS2:ERG could be a prognostic biomarker for aggressive prostate cancer.

Following Tomlins' pioneering discovery, subsequent research has identified a host of similar ETS family gene fusions. Other oncogenic ETS transcription factors, for example, ETV4 [39], ETV5 [40], and ELK4 [41], have been identified as additional fusion partners for TMPRSS2. Other unique 5′ fusion partner genes to ETS family members have also been identified, such as SLC45A3, HERV-K_22q11.23, HNRPA2B1, and C15ORF21 in fusion with ETV1 [42], KLK2 and CANT1 in fusion with ETV4 [43], and SLC45A3:ETV5 [40].

For ETS fusion-negative prostate cancers subtypes, novel gene fusions have also been identified, including SLC45A3:BRAF, ESRP1:RAF1, SLC45A3:BRAF, ESRP1:RAF1 [44], C15orf21:Myc [45], EPB41:BRAF [46], and TMEM79:SMG5 [47].

#### 3.2.2. Noncoding RNAs

In addition to gene fusions, RNA-Seq also enables discovery of new noncoding RNAs (ncRNAs) with the potential to serve as cancer markers. Transcriptome sequencing of a prostate cancer cohort has identified an unannotated ncRNA PCAT-1 as a transcriptional repressor linked to PCa progression [48]. RNA- Seq was also applied to identify differentially expressed microRNAs (e.g., miR-16, miR-34a, miR-126*, miR-145, and miR-205) associated with metastatic prostate cancer [49]. These findings establish the utility of RNA-Seq to identify disease-associated ncRNAs that could provide potential biomarkers or therapeutic targets.

### 3.3. ChIP-Seq

Another application of NGS capitalizes on the ability to analyze protein-DNA interactions, as for ChIP-Seq. ChIP-Seq provides clear indications of transcription factor binding sites (TFBSs) at high resolution. It is also well suited for detecting patterns of modified histones in a genome-wide manner [78].

Much of gene regulation occurs at the level of transcriptional control. In addition, aberrant histone modifications (methylation or acetylation) are also associated with cancer. Therefore experimental identification of TFBSs or histone modifications has been an area of high interest. In traditional ChIP-chip approaches, DNA associated with a transcription factor or histone modification of interest is first selectively enriched by chromatin immunoprecipitation, followed by probing on DNA microarrays. In contrast to ChIP-chip, ChIP-Seq uses NGS instead of custom-designed arrays to identify precipitated DNA fragments, thus yielding more unbiased and sensitive information about target regions.

Many efforts have employed ChIP-Seq approaches to characterize transcriptional occupancy of AR, and many novel translational partners of AR have been identified. Using ChIP-Seq, Chng et al. [50] performed global analysis of AR and ERG binding sites. They revealed that ERG promotes prostate cancer progression by working together with transcriptional corepressors including HDACs and EZH2. Zhang et al. [51] developed a comotif scanning program called CENTDIST and applied it on an AR ChIP-Seq dataset from a prostate cancer cell line. They correctly predicted all known co-TFs of AR as well as discovered AP4 as a novel AR co-TF. Little et al. [53] used genome-wide ChIP-Seq to study Runx2 occupancy in prostate cancer cells. They suggested novel role of Runx2a in regulating secretion invasiveness and membrane secretion. Tan et al. [54] showed that NKX3-1 colocalizes with AR and proposed a critical transcriptional regulatory network between NKX3-1, AR, and the RAB GTPase signaling pathway in prostate cancer. Urbanucci et al. [79] identified AR-binding sites and demonstrated that the overexpression of AR enhances the receptor binding to chromatin in CRPC. By comparing nucleosome occupancy maps using nucleosome-resolution H3K4me2 ChIP-Seq, He et al. [52] found that nucleosome occupancy changes can predict transcription factor cistromes. This approach also correctly predicted the binding of two factors, POU2F1 and NKX3-1. By high-resolution mapping of intra- and interchromosome interactions, Rickman et al. [80] demonstrated that ERG binding is enriched in hotspots of differential chromatin interaction. Their result indicated that ERG overexpression is capable of inducing changes in chromatin structures.

Taken together, these studies have provided a complete regulatory landscape in prostate cancer.

### 3.4. Methyl-Seq

Another fertile area for NGS involves assessment of the genome-wide methylation status of DNA. Methylation of cytosine residues in DNA is known to silence parts of the genome by inducing chromatin condensation. DNA hypermethylation probably remains most stable and abundant epigenetic marker.

Several DNA methylation markers have been identified in prostate cancer. The most extensively studied one is CpG island hypermethylation of glutathione-S-transferase P (GSTP1) promoter DNA, resulting in the loss of GSTP1 expression [81]. Today GSTP1 hypermethylation is most frequently evaluated as diagnostic biomarker for prostate cancer. It is also an adverse prognostic marker that predicts relapse of patients following radical prostatectomy [82].

With the aid of NGS technologies, genome-wide mapping of methylated cytosine patterns in cancer cells become feasible. Based on established epigenetics methods, there are emerging next-generation sequencing applications for the interrogation of methylation patterns, including methylation-dependent immunoprecipitation sequencing (MeDIP-Seq) [83], cytosine methylome sequencing (MethylC-Seq) [84], reduced representation bisulfite sequencing (RRBS-Seq) [85], methyl-binding protein sequencing (MBP-Seq) [86], and methylation sequencing (Methyl-Seq) [87]. A number of aberrant methylation profiles have been developed so far and are being evaluated as potential markers for early diagnosis and risk assessment. As an example, using MethylPlex-Seq, Kim et al. [55] mapped the global DNA methylation patterns in prostate tissues and revealed distinct patterns of promoter methylation around transcription start sites. The comprehensive methylome map will further our understanding of epigenetic regulation in prostate cancer progression.

## 4. PCa Biomarkers in Combinations

Diagnostic and prognostic markers with relevance to PCa are routinely identified. Nevertheless, we should stay aware that candidate markers obtained are often irreproducible from experiment to experiment and very few molecules will make it to the routine clinical practice. Cancer is a nonlinear dynamic system that involves the interaction of many biological components and is not driven by individual causative mutations. In most cases, no single biomarker is likely to dictate diagnosis or prognosis success. Consequently, the future of cancer diagnosis and prognosis might rely on the combination of a panel of markers. Kattan and associates [88] have established a prognostic model that incorporates serum TGF-*β*1 and IL-6R for prediction of recurrence following radical prostatectomy. This combination shows increased predictive accuracy from 75 to 84%. Furthermore, a predictive model incorporating GSTP1, retinoic acid receptor *β*2 (RAR *β*2), and adenomatous polyposis coli (APC) has been assessed. But no increased diagnostic accuracy was shown compared with PSA level alone [89]. More recently a panel of markers, PCA3, serum PSA level, and % free PSA show improved predictive value compared with PSA level and % free PSA. Again the clinical utility of these combinations needs to be evaluated in large-scale studies.

## 5. PCa Biomarkers at Pathway Level

Recently, an importance of pathway analysis has been emphasized in the study of cancer biomarkers. Pathway-based approach allows biologists to detect modest expression changes of functionally important genes that would be missed in expression-alone analysis. In addition, this approach enables the incorporation of previously acquired biological knowledge and makes a more biology-driven analysis of genomics data. Pathway analysis typically correlates a given set of molecular changes (e.g., differential expression, mutation, and copy number variation data) by projecting them onto well-characterized biological pathways. A number of curated databases are available for canonical signaling and metabolic pathways, such as Kyoto Encyclopedia of Genes and Genomes (KEGG, http://www.genome.jp/kegg/), Molecular Signatures Database (MSigDB), IngenuityPathway Analysis (IPA, http://www.ingenuity.com/), GeneGO by MetaCore (http://www.genego.com/), and Gene Set Enrichment Analysis (GSEA, http://www.broadinstitute.org/gsea/). Enrichment of pathways can be evaluated by overrepresentation statistics. The overall flowchart of the proposed pathway-based biomarker approach is illustrated in Figure 1. Using this pipeline, the pathways enriched with aberrations are identified and then proposed to be the potential candidate markers. 

Some impressive progress has been made to identify pathways with relevance to the pathophysiology of prostate cancer. Rhodes et al. [90] were of the first to perform pathway analysis of the microarray expression datasets. By meta-analysis of 4 independent microarray datasets, they generated a cohort of genes that were commonly deregulated in PCa. The authors then mapped the identified deregulated genes to functional annotations and pinpointed polyamine and purine biosynthesis as critical pathway altered in PCa. Activation of Wnt signaling pathway was reported to be key pathways defining the poor PCa outcome group [91]. A comparison of castration-resistant and castration-sensitive pairs of tumor lines highlighted the Wnt pathway as potentially contributing to castration resistance [37]. Using a similar pathway-based approach, Wang et al. found Endothelin-1/EDNRA transactivation of the EGFR a putative novel PCa related pathway [92]. More recently, an integrative analysis of genomic changes revealed the role of the PI3K, RAS/RAF, and AR pathways in metastatic prostate cancers [6]. The above insights provide a blueprint for the design of novel pathway inhibitors in targeted therapies for prostate cancer. 

Thus far the pathway-based approach holds great promise for cancer prediction. However, the known pathways correspond merely to a small fraction of somatic alterations. The alterations that have not been assigned to a definitive pathway undermine the basis for a strictly pathway-centric marker discovery. In addition, the cross-talk between different signaling pathways further complicated the pathway-based analysis. Thus, biomarker discovery has to shift toward an integrative network-based approach that accounts more extensive genomic alteration.

## 6. PCa-Specific Databases

High throughput research in PCa has led to vast amounts of comprehensive datasets. There has been a growing desire to integrate specific data types into a centralized database and make them publicly available. Considerable efforts were undertaken and to date various national, multicenter, and institutional databases in the context of prostate cancer research are available. Prostate gene database (PGDB, http://www.urogene.org/pgdb/) is a curated database on genes or genomic loci related to human prostate and prostatic diseases [93]. Another database, prostate expression database (PEDB, http://www.pedb.org/) is a curated database that contains tools for analyzing prostate gene expression in both cancerous and normal conditions [94]. Dragon database of genes associated with prostate cancer (DDPC, http://cbrc.kaust.edu.sa/ddpc/) [95] is an integrated knowledge database that provides a multitude of information related to PCa and PCa-related genes. ChromSorter [96] collects PCa chromosomal regions associated with human prostate cancer. PCaMDB is a genotype-phenotype database that collects prostate cancer related gene and protein variants from published literatures. These specific databases tend to include large numbers of patients from different geographic regions. Their generalizability and statistical power offer researchers a unique opportunity to conduct prostate cancer research in various areas.

## 7. Translational Bioinformatics in PCa: A Future Direction

**Figure 2:**
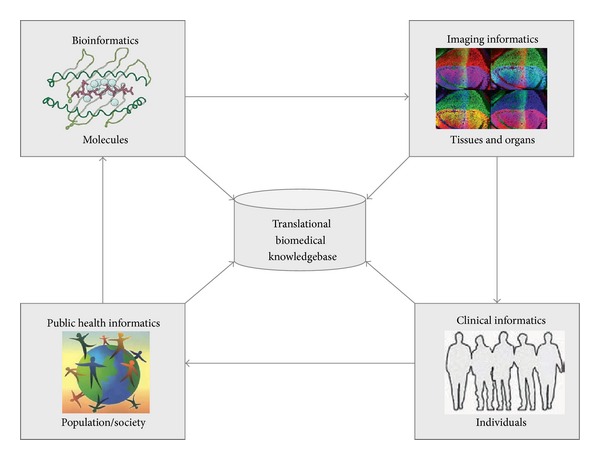
Translational bioinformatics bridges knowledge from molecules to populations. Four subdisciplines of translational bioinformatics and their respective focus areas are depicted in boxes. The success of translational bioinformatics will enable a complete information logistics chain from single molecules to the entire human population and thus link innovations from bench to bedside.

Recent advances in NGS technologies have resulted in huge sequence datasets. This poses a tremendous challenge for the emerging field of translational bioinformatics. Translational bioinformatics, by definition, is the development of storage, analytic, and interpretive methods to optimize the translation from bench (laboratory-based genomic discoveries) to bedside (evidence-based clinical applications). The aim of translational bioinformatics is to combine the innovations and resources across the entire spectrum of translational medicine towards the betterment of human health. To achieve this goal, the fundamental aspects of bioinformatics (e.g., bioinformatics, imaging informatics, clinical informatics, and public health informatics) need to be integrated (Figure 2).

As the first aspect, bioinformatics is concerned with applying computational approaches to comprehend the intricate biological details elucidating molecular and cellular processes of cancer. Imaging informatics is focused on what happens at the level of tissues and organs, and informatics techniques are used for image interpretation. Clinical bioinformatics focuses on data from individual patients. It is oriented to provide the technical infrastructure to understand clinical risk factors and differential response to treatment at the individual levels. As for public health informatics, the stratified population of patients is at the center of interest. Informatics solutions are required to study shared genetic, environmental, and life style risk factors on a population level. In order to achieve the technical and semantic interoperability of multidimensional data, fundamental issues in information exchange and repository are to be addressed.

## 8. Future Perspectives

The prospects for NGS-based biomarkers are excellent. However, compared with array-based studies, real in-depth NGS is still costly and also the analysis pipeline is less established, thus challenging the use of NGS. A survey in the GEO indicated that NGS is currently finding modest application in the identification of PCa markers. Table 1 listed the number of published GEO series on human PCa using NGS versus microarrays. Therefore, further work is still required before NGS can be routinely used in the clinic. Issues regarding data management, data integration, and biological variation will have to be tackled. 

### 8.1. NGS in the Cloud

**Figure 3:**
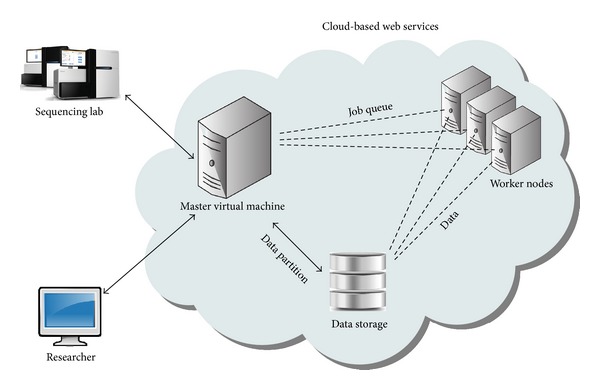
Schematic of the cloud-based NGS analysis. Local computers allocate the cloud-based web services over the internet. Web services comprised a cluster of virtual machines (one master node and a chosen number of worker nodes). Input data are transferred to the cloud storage and the program code driving the computation is uploaded to master nodes, by which worker nodes are provisioned. Each worker node downloads reads from the storage and run computation independently. The final result is stored and meanwhile transferred to the local client computer and the job completes.

The dramatic increase in sequencer output has outpaced the improvements in computational infrastructure necessary to process the huge volumes of data. Fortunately, an alternative computing architecture, cloud computing, has recently emerged, which provides fast and cost-effective solutions to the analysis of large-scale sequence data. In cloud computing, high parallel tasks are run on a computer cluster through a virtual operating system (or “cloud”). Underlying the clouds are compact and virtualized virtual machines (VMs) hosting computation-intensive applications from distributed users. Cloud computing allows users to “rent” processing power and storage virtually on their demand and pay for what they use. There has been considerable enthusiasm in the bioinformatics community for use of cloud computing services.  Figure 3 shows a schematic drawing of the cloud-based NGS analysis. 

Recently exploratory efforts have been made in cloud-based DNA sequence storage. O'Connor et al. [97] created SeqWare Query Engine using cloud computing technologies to support databasing and query of information from thousands of genomes. BaseSpace is a scalable cloud-computing platform for all of Illumina's sequencing systems. After a sequencing run is completed, data from sequencing instruments is automatically uploaded to BaseSpace for analysis and storage. DNAnexus, a company specialized in Cloud-based DNA data analysis, has leveraged the storage capacity of Google Cloud to provide high-performance storage for NGS data.

 Some initiatives have utilized preconfigured software on such cloud systems to process and analyze NGS data.  Table 3 summarizes some tools that are currently available for sequence alignment, short read mapping, single nucleotide polymorphism (SNP) identification, and RNA expression analysis, amongst others. 

Although cloud computing seems quite attractive, there are also issues that are yet to be resolved. The most significant concerns pertain to information security and bandwidth limitation. Transferring massive amounts of data (on the order of petabytes) to the cloud may be time consuming and prohibitively expensive. For most sequencing centers that require substantial data movement on a regular basis, cloud computing currently does not make economic sense. While it is clear that cloud computing has great potential for research purposes, for small labs and clinical applications using benchtop genome sequencers of limited throughput, like MiSeq, PGM, and Proton, cloud computing does have some practical utility. 

### 8.2. Biomarker Discovery Using Systems Biology Approach

NGS makes it possible to generate multiple types of genomic alterations, including mutations, gene fusions, copy number alterations, and epigenetic changes simultaneously in a single test. Integration of these genomic, transcriptomic, interactomic, and epigenomic pieces of information is essential to infer the underlying mechanisms in prostate cancer development. The challenge ahead will be developing a comprehensive approach that could be analysed across these complementary data, looking for an ideal combination of biomarker signatures.

Consequently, the future of biomarker discovery will rely on a systems biology approach. One of the most fascinating fields in this regard is the network-based approaches to biomarker discovery, which integrate a large and heterogeneous dataset into interactive networks. In such networks, molecular components and interactions between them are represented as nodes and edges, respectively. The knowledge extracted from different types of networks can assist discovery of novel biomarkers, for example, functional pathways, processes,or subnetworks, for improved diagnostic, prognostic, and drug response prediction. Network-based discovery framework has already been reported in several types of cancers [98–102] including PCa [103, 104]. In a pioneering study, Jin et al. [103] built up a prostate-cancer-related network (PCRN) by searching the interactions among identified molecules related to prostate cancer. The network biomarkers derived from the network display high-performances in PCa patient classification. 

As the field high-throughput technologies continues to develop, we will expect enhancing cooperation among different disciplines in translational bioinformatics, such as bioinformatics, imaging informatics, clinical informatics, and public health informatics. Notably, the heterogeneous data types coming from various informatics platforms are pushing for developing standards for data exchange across specialized domains.

### 8.3. Personalized Biomarkers

The recent breakthroughs in NGS also promise to facilitate the area of “personalised” biomarkers. Prostate cancer is highly heterogeneous among individuals. Current evidence indicates that the inter-individual heterogeneity arises from genetical environmental and lifestyle factors. By deciphering the genetic make-up of prostate tumors, NGS may facilitate patient stratification for targeted therapies and therefore assist tailoring the best treatment to the right patient. It is envisioned that personalized therapy will become part of clinical practice for prostate cancer in the near future.

## 9. Conclusions

Technological advances in NGS have increased our knowledge in molecular basis of PCa. However the translation of multiple molecular markers into the clinical realm is in its early stages. The full application of translational bioinformatics in PCa diagnosis and prognosis requires collaborative efforts between multiple disciplines. We can envision that the cloud-supported translational bioinformatics endeavours will promote faster breakthroughs in the diagnosis and prognosis of prostate cancer.

## Figures and Tables

**Table 1:** Number of PCa-associated GEO series generated by microarray and NGS.

Methodology	Gene expression profiling	Noncoding RNA profiling	Genome binding/occupancy profiling	Genome methylation profiling	Genome variation profiling
Microarray	266	34	17	21	35
NGS	11	1	18	2	2

**Table 2:** Summary of NGS-based studies on prostate cancer.

Discoveries	Method	References
Copy number loss of MTAP, CDKN2, and ARF genes	DNA-Seq	[34]
Somatic mutations in MTOR, BRCA2, ARHGEF12, and CHD5 genes		[11]
NCOA2, p300, the AR corepressor NRIP1/RIP140, and NCOR2/SMRT		[6]
Somatic mutations in SPOP, FOXA1, and MED12		[35]
Somatic mutations in MLL2 and FOXA1		[36]
Somatic mutations in TP53, DLK2, GPC6, and SDF4.		[37]

TMPRSS2:ERG, TMPRSS2:ETV1	RNA-Seq	[38]
TMPRSS2:ETV4		[39]
TMPRSS2:ETV5, SLC45A3:ETV5		[40]
TMPRSS2:ELK4		[41]
SLC45A3:ETV1, HERV-K_22q11.23:ETV1, HNRPA2B1:ETV1, and C15ORF21:ETV1		[42]
KLK2:ETV4 and CANT1:ETV4		[43]
SLC45A3:BRAF or ESRP1:RAF1		[44]
C15orf21:Myc		[45]
EPB41:BRAF		[46]
TMEM79:SMG5		[47]
Differential expression of PCAT-1		[48]
Differential expression of miR-16, miR-34a, miR-126*, miR-145, and miR-205		[49]

HDACs and EZH2 work as ERG corepressors	Chip-Seq	[50]
AP4 as a novel co-TF of AR		[51]
POU2F1 and NKX3-1		[52]
Runx2a regulates secretion invasiveness and membrane secretion		[53]
A novel transcriptional regulatory network between NKX3-1, AR, and the RAB GTPase signaling pathway		[54]

Distinct patterns of promoter methylation around transcription start sites	Methyl-Seq	[55]

**Table 3:** The cloud computing software for NGS data analysis.

Software	Website	Description	References
Crossbow	http://bowtie-bio.sourceforge.net/crossbow/	Read mapping and SNP calling	[56]
CloudBurst	http://cloudburst-bio.sourceforge.net/	Reference-based read mapping	[57]
Contrail	http://contrail-bio.sourceforge.net/	De novo read assembly	[58]
Cloud-MAQ	http://sourceforge.net/projects/cloud-maq/	Read mapping and assembly	[59]
Bioscope	http://www.lifescopecloud.com/	Reference-based read mapping	[60]
GeneSifter	http://www.geospiza.com/Products/AnalysisEdition.shtml	Customer oriented NGS data analysis services	[61]
CloudAligner	http://sourceforge.net/projects/cloudaligner/	Read mapping	[62]
Roundup	http://rodeo.med.harvard.edu/tools/roundup	Optimized computation for comparative genomics	[63]
PeakRanger	http://www.modencode.org/software/ranger/	Peak caller for ChIP-Seq data	[64]
Myrna	http://bowtie-bio.sf.net/myrna/	Differential expression analysis for RNA-Seq data	[65]
ArrayExpressHTS	http://www.ebi.ac.uk/Tools/rwiki/	RNA-Seq data processing and quality assessment	[66]
SeqMapreduce	Not available	Read mapping	[67]
BaseSpace	https://basespace.illumina.com/home/index		
